# Giant retroperitoneal well-differentiated liposarcoma presenting in emergency with intestinal occlusion: Case report and review of the literature

**DOI:** 10.1016/j.ijscr.2022.107152

**Published:** 2022-05-03

**Authors:** Giuseppe Evola, Riccardo Schillaci, Martina Reina, Giovambattista Caruso, Maria D'Angelo, Giuseppe Angelo Reina

**Affiliations:** a General and Emergency Surgery Department, Garibaldi Hospital, Catania, Italy; b General Surgery Department, San Salvatore Hospital, Paternò (Catania), Italy

**Keywords:** Retroperitoneal, Well-differentiated, Liposarcoma, Sarcoma, Surgery, Case report

## Abstract

**Introduction and importance:**

Liposarcoma (LPS) represents the most common type of retroperitoneal sarcoma (RPS) and can be classified into four subtypes. Preoperative diagnosis of retroperitoneal liposarcoma (RLPS) is a challenge because of its *late* and nonspecific *clinical presentation*. Imaging may be helpful for determining the correct diagnosis. Surgery represents a potentially curative treatment of RLPS.

**Case presentation:**

A 55-year-old Caucasian female presented to the Emergency Department with a two-day history of abdominal pain, abdominal distension, inability to pass gas or stool, nausea, vomiting and lipothymia. Abdominal examination revealed abdominal distention, abdominal pain without obvious muscle guarding and a giant non-tender mass. Laboratory tests reported neutrophilic leukocytosis and anemia. Abdominal contrast-enhanced computed tomography (CECT) showed a heterogeneous and hypodense giant retroperitoneal mass compressing and displacing the surrounding organs and vessels. The patient underwent excision of a giant retroperitoneal mass. The postoperative course of the patient was uneventful.

**Clinical discussion:**

RLPS is a malignant neoplasm that can slowly grow to enormous size with possible involvement of adjacent organs and vessels; it may recur locally and has a minimal capacity to metastasize. Preoperative diagnosis and staging of RLPS are important to establish appropriate management and prognosis. Surgery represents the gold standard for non-metastatic RLPS treatment.

**Conclusion:**

RLPS is a rare malignant neoplasm generally difficult to detect early due to its *late* and nonspecific *clinical presentation*. CECT represents the most commonly used modality for diagnosis, staging and preoperative evaluation. Surgery represents the appropriate treatment of non-metastatic RLPS.

## Highlights

• Liposarcoma is the most common type of retroperitoneal sarcoma.
• Early diagnosis of retroperitoneal liposarcoma is a challenge due to its nonspecific *clinical presentation*.
• Computed tomography and magnetic resonance imaging are the most relevant imaging modality for diagnosis
• Surgery represents a potentially curative treatment of retroperitoneal liposarcoma.

## 1 Introduction

Retroperitoneal sarcomas (RPSs) are rare malignant neoplasms that account for approximately 12–15% of all soft tissue sarcomas, with a mean incidence of 3–4/million [1]. Liposarcoma (LPS) represents the most common type of RPS, followed by leiomyosarcoma and malignant fibrous histiocytoma, and alone encompasses 0.07–0.2% of all neoplasms [2]. LPS originates from fat and can be classified into four subtypes, representing well-differentiated liposarcoma (WDLPS) the most common subtype. Retroperitoneal liposarcoma (RLPS) is generally difficult to detect early due to its *late* and nonspecific *clinical presentation*. Preoperative diagnosis and staging are important to establish appropriate management and prognosis. A rare case of giant RLPS, presenting in emergency with intestinal occlusion, is presented with review of the literature in accordance with SCARE 2020 criteria [3]. The purpose of this case report is to remember that early diagnosis of RLPS is difficult and surgery represents a potentially curative treatment.

## 2 Presentation of case

**Fig. 1:**
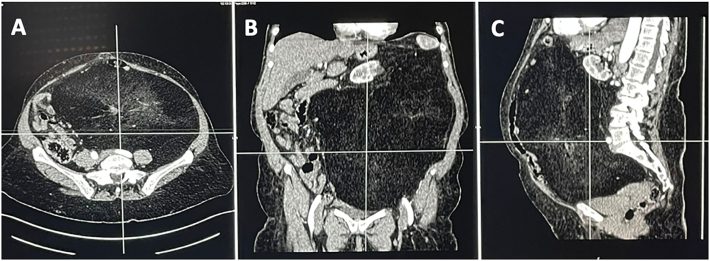
A, B, C. Abdominal contrast-enhanced computed tomography (CECT) showing a heterogeneous and hypodense (fat density) giant retroperitoneal mass, measuring 36 × 32 × 28 cm, compressing and displacing the surrounding intra- and retro-peritoneal organs and vessels (A axial view, B coronal view, C sagittal view).

**Fig. 2:**
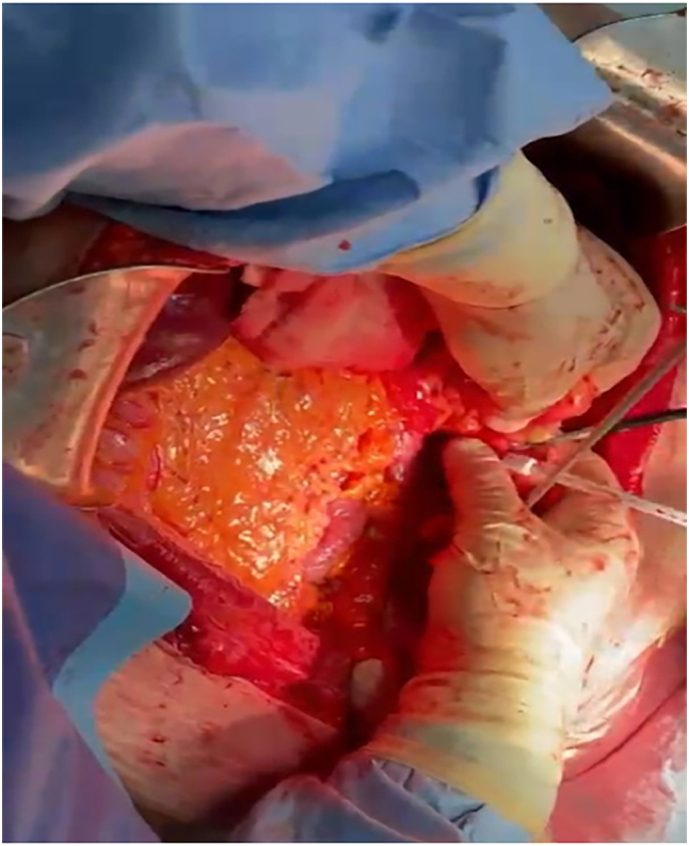
Giant retroperitoneal fatty mass compressing and displacing the intestine in the right side of the abdominal cavity: operative findings.

**Fig. 3:**
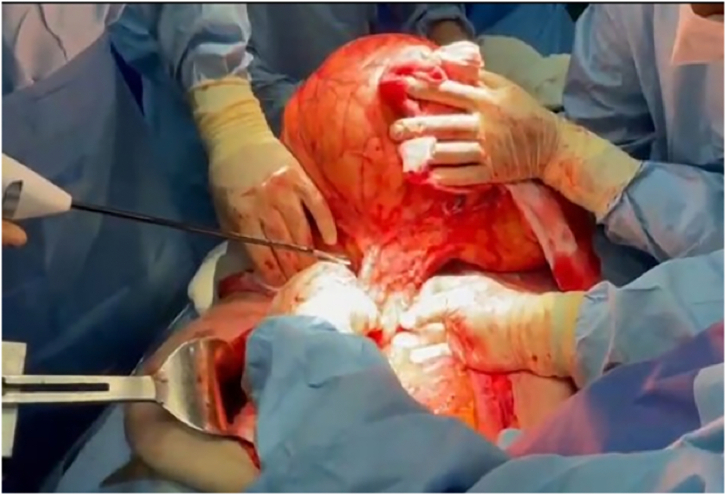
Total excision of the giant retroperitoneal fatty mass without the need to remove other organs: operative findings.

**Fig. 4:**
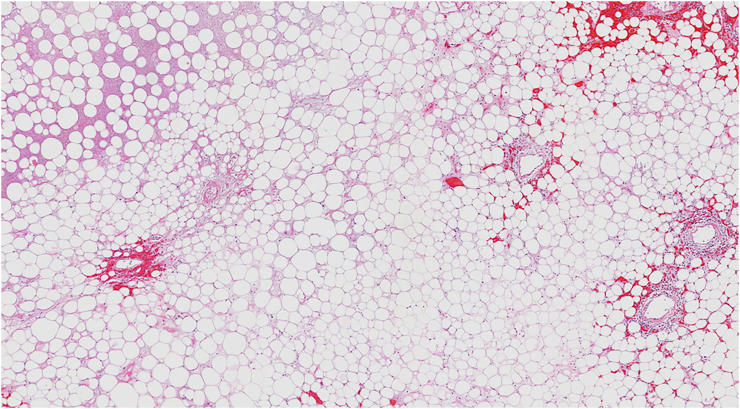
Photomicrograph section of retroperitoneal well-differentiated liposarcoma (haematoxylin and eosin, original magnification ×40).

A 55-year-old Caucasian female, with a medical history of hypertension, presented to the Emergency Department with a two-day history of abdominal pain, abdominal distension, inability to pass gas or stool, nausea, vomiting and lipothymia. She was pale, hypotensive, tachycardic and tachypnoic. Vital signs were blood pressure 90/45 mmHg, pulse 115 bpm, respiratory rate 24 per minute, oxygen saturation 91% in ambient air and temperature of 37, 2 °C. She wasn't taking any drug, referred habit on smoking but denied alcohol consumption; her familial medical history was normal. She was employed by profession, married and of medium socio-economic status. Physical examination of the abdomen revealed abdominal distention, abdominal pain on deep palpation of the right quadrants without obvious muscle guarding and a giant non-tender mass in the left and in the midline quadrants. Laboratory tests reported neutrophilic leukocytosis (WBC 18.800 × 10^3^/μL) and anemia (hemoglobin 7.5 g/dl). The patient was initially managed with fluids, transfusion of three units of packed red blood cells, intravenous broad-spectrum antibiotics and bowel rest. Abdominal contrast-enhanced computed tomography (CECT) showed a heterogeneous and hypodense giant retroperitoneal mass, measuring 36 × 32 × 28 cm, compressing and displacing the surrounding intra- and retro-peritoneal organs and vessels and suspected of retroperitoneal soft tissue sarcoma (Fig. 1A, B, C). The patient, after understanding the severity of her medical condition and accepting surgery, was taken emergently to the operating room by experienced general surgeons for explorative laparotomy under general anesthesia. The patient was placed in the supine position on the operating table: intraoperatively the giant retroperitoneal fatty mass was found not arising from major solid *organs*, compressing and displacing the intestine and the left kidney in the right side of the abdominal cavity (Fig. 2). A total excision of the giant retroperitoneal mass was performed without the need to remove other organs (Fig. 3), a pelvic drain was placed. Patient was given an IV injection of Amoxicillin/Clavulanate 2 g twice daily for five days. The postoperative course was uneventful: the patient was discharged on the 5th postoperative day, after removal of the abdominal drain, in a stable condition. The surgical specimen consisted of a voluminous retroperitoneal fatty mass measuring 36 × 32 × 28 cm and weighing 21 kg. Histopathological examination revealed a giant WDLPS (*French National Federation of Cancer Centers grading* system: Histologic grade 1, Tumor differentiation: score 1; Mitotic count: score 1; tumor necrosis: score 0) (Fig. 4). The patient tolered the advice provided, was referred to Oncology Department and after a follow-up of twelve months is asymptomatic.

## 3 Discussion

RPSs are rare neoplasms (12–15% of all sarcomas) [1], accounting for 1–2% of all solid malignancies in adults [4]. LPS represents the most common type of sarcoma arising in the retroperitoneum and generally originates in the perirenal fat. LPS, according to the 2013 WHO classification of soft tissue and bone tumors, can be classified into four histologic subtypes: well-differentiated liposarcoma (WDLPS), dedifferentiated liposarcoma, myxoid/round cell liposarcoma and pleomorphic liposarcoma. WDLPS is the most common RLPS (accounting for 40–45% of all LPS) [5] and can also be classified in lipomatous, sclerotic and inflammatory LPS [5]. The peak incidence of RLPS is in the sixth and seventh decades without sex or racial predilection [1]. The clinical behavior of LPS ranges from indolent nonmetastasizing disease to aggressive subtypes that can recur and metastasize rapidly [6]. Retroperitoneum (19%) represents the second most common site of LPS origin after the extremities (52%) [7]. Most of LPSs are malignant from their inception, only few LPSs originate from benign lipomas. WDLPS can slowly grow to enormous size, due to the large potential space of the retroperitoneum, with possible involvement of adjacent organs and vessels; it may recur locally and has a minimal capacity to metastasize. However, dedifferentiation and trans-differentiation of WDLPS may lead to its progression (15% of cases) into high-grade tumors [1]. RPLSs are frequently incidental findings in the work-up for no-related symptoms or diseases and can grow to very large size (approximately 50% of RPLSs are >20 cm when diagnosed) [8] before inducing nonspecific symptoms and signs like as abdominal pain, flank/back pain, abdominal distention, early satiety and a palpable abdominal mass. Local invasion or compression of the retroperitoneal structures may present as neurological, musculoskeletal or obstructive urinary/bowel symptoms [1]. LPS weighing over 20 kg, as in our case, is defined as “giant liposarcoma”. The main differential diagnosis of RPLS includes adrenal/renal/pancreatic tumors, advanced gastrointestinal carcinomas, lymphomas, lymphangiomas, retroperitoneal fibrosis and metastatic carcinomas. Different imaging studies are useful for diagnosis of RPLS although CECT and magnetic resonance imaging (MRI) remain the most relevant imaging modality for diagnosis. Abdominal radiography indicates the displacement of bowel and an altered intestinal aeration. Ultrasound may reveal a multilobulated retroperitoneal hyperechoic mass. Doppler ultrasound can assess the patency of the femoral/iliac vessels and of the inferior caval vein. Contrast-enhanced studies of the gastrointestinal tract may show displacement of the stomach and bowel [1]. On CT scan and MRI, WDLPS appears as a predominantly adipose soft tissue mass with smooth margins, a lobular contour, septations thicker than 2 mm and small foci (<2 cm) of nodular tissue. Septations and nodular areas in WDLPS show hyperintense character on T2-W1 MRI [9]. CECT represents, as in our case, the most commonly used modality for diagnosis, staging and preoperative evaluation of WDLPS. More recently PET-FDG imaging has been used in an effort to assess the tumor grading, staging as well as to evaluate patients for tumor recurrence [1]. Currently no consensus exists regarding the need of image-guided core needle biopsies of a suspected RPLS before treatment. Biopsy must be recommended unless imaging is pathognomonic for WDLPS and no preoperative treatment is planned for unresectable or metastatic tumors. Accurate staging is important as it facilitates determination of appropriate surgery, establishes prognosis, and provides a guide for adjunctive therapy. Prognosis of RPLS depends on age, anatomical location, size, histological subtype, resection margins and distant metastasis of the tumor [10]. Surgery represents the gold standard treatment of non-metastatic RPLS. The aim of surgical resection should be to achieve a macroscopic complete R0/R1 resection. A complete resection of the tumor with negative microscopic margins and en bloc removal of involved adjacent structures has been shown to improve overall survival: in a previous study of 500 RPLSs, the median survival of patients after complete resection was 103 months in contrast to 18 months after incomplete resection [11]. The extent of surgical resection is still debatable: an extended resection with en block resection of contiguous uninvolved organs is advocated to reduce local recurrence rates. However retrospective studies have shown that extended resection lowers the risk of local recurrence but its effect on overall survival remains unclear [12]. Causes for RPLS nonresectability are metastases and infiltration of vital structures [13]. Neoadjuvant therapies are applicable in the setting of advanced disease because of RLPS is radiosensitive but modestly chemosensitive. A large retrospective review of advanced RLPSs did not show impact of chemotherapy on progression-free or overall survival [14]. Preoperative radiotherapy can decrease risk of local recurrence [15] but there has not been a demonstrably clear improvement in overall disease-free survival [16]. Unresectable RPLS that becomes resectable after neoadjuvant therapies should be surgically removed. No trial shows a benefit from adjuvant chemotherapy for RLPSs. Adjuvant radiotherapy may improve local control, specifically with involved margins or high-grade tumors. RLPS generally recurs within 6–24 months after surgery. Follow-up imaging is usually performed with CT or MRI and should be at least 10 years or even indefinite: patients with WDLPS who have been successfully resected should have a follow-up imaging every 3–6 months for the first five years, then annually [17]. Local recurrences of RLPS can often be misinterpreted like as post-operative scarring/fibrosis and a complete excision of the lesion will account to a better outcome. The five-year survival rate of WDLPS is approximately 90% [10], mortality is usually due to its uncontrolled local recurrence.

## 4 Conclusion

Retroperitoneal WDLPS is a rare malignant tumor characterized by slow progressive growth, local invasion and local recurrence after surgery but does not exhibit metastatic potential. Preoperative diagnosis and staging is essential for optimal management of RLPS. Surgery represents the mainstay of treatment for non-metastatic RLPS.

## Sources of funding

This research did not receive any specific grant from funding agencies in the public, commercial, or not-for-profit sectors.

## Ethical approval

Ethical approval has been exempted by our institution because this is a case report and no new studies or new techniques were carried out.

## Consent

Written informed consent was obtained from the patient, for publication of this case report and accompanying images. A copy of the written consent is available for review by the Editor-in-Chief of this journal on request.

## Author's contribution

Giuseppe Evola: Drafting the manuscript, literature research.

Riccardo Schillaci: Operated on the patient, drafting the manuscript.

Martina Reina: Drafting the manuscript and literature research.

Giovambattista Caruso: Operated on the patient, drafting the manuscript.

Maria D'Angelo: Drafting the manuscript, literature research.

Giuseppe Angelo Reina: Operated on the patient, revising the manuscript.

## Registration of research studies

Not applicable.

## Guarantor

Giuseppe Evola.

## Provenance and peer review

Not commissioned, externally peer-reviewed.

## Declaration of competing interest

All the authors certify that there is no conflict of interest regarding the material discussed in the manuscript.

## References

[1] Vijay A., Ram L. (2015 Apr). Retroperitoneal liposarcoma: a comprehensive review. Am. J. Clin. Oncol..

[2] Echenique-Elizondo M., Amondarain-Arratíbel J.A. (2005 May). Liposarcoma retroperitoneal gigante [Giant retroperitoneal liposarcoma]. Cir. Esp..

[3] Agha R.A., Franchi T., Sohrabi C., Mathew G., Kervan A., for the SCARE Group (2020). The SCARE 2020 guideline: updating consensus Surgical CAse REport (SCARE) guidelines. Int. J. Surg..

[4] Francis I.R., Cohan R.H., Varma D.G., Sondak V.K. (2005 Aug 23). Retroperitoneal sarcomas. Cancer Imaging.

[5] Yang L., Chen S., Luo P., Yan W., Wang C. (2020 Jan 1). Liposarcoma: advances in cellular and molecular genetics alterations and corresponding clinical treatment. J. Cancer.

[6] O'Regan K.N., Jagannathan J., Krajewski K. (2011 Jul). Imaging of liposarcoma: classification, patterns of tumor recurrence, and response to treatment. AJR Am. J. Roentgenol..

[7] Xiao J., Liu J., Chen M., Liu W., He X. (2021 Apr). Diagnosis and prognosis of retroperitoneal liposarcoma: a single Asian center cohort of 57 cases. J. Oncol..

[8] Chouairy C.J., Abdul-Karim F.W., MacLennan G.T. (2007). Retroperitoneal liposarcoma. J. Urol..

[9] Teniola O., Wang K.Y., Wang W.L., Tseng W.W., Amini B. (2018). Imaging of liposarcomas for clinicians: characteristic features and differential considerations. J. Surg. Oncol..

[10] Xu C., Ma Z., Zhang H., Yu J., Chen S. (2020 Oct). Giant retroperitoneal liposarcoma with a maximum diameter of 37 cm: a case report and review of literature. Ann. Transl. Med..

[11] Lewis J.J., Leung D., Woodruff J.M., Brennan M.F. (1998). Retroperitoneal soft-tissue sarcoma: analysis of 500 patients treated and followed at a single institution. Ann. Surg..

[12] Gronchi A., Vullo S.Lo, Fiore M. (2009). Aggressive surgical policies in a retrospectively reviewed single-institution case series of retroperitoneal soft tissue sarcoma patients. J. Clin. Oncol..

[13] Bonvalot S., Raut C.P., Pollock R.E. (2012 Sep). Technical considerations in surgery for retroperitoneal sarcomas: position paper from E-surge, a master class in sarcoma surgery, and EORTC-STBSG. Ann. Surg. Oncol..

[14] Italiano A., Toulmonde M., Cioffi A. (2012). Advanced well-differentiated/dedifferentiated liposarcomas: role of chemotherapy and survival. Ann. Oncol..

[15] Sampath S., Hitchcock Y.J., Shrieve D.C., Randall R.L., Schultheiss T.E., Wong J.Y. (2010). Radiotherapy and extent of surgical resection in retroperitoneal soft-tissue sarcoma: multi-institutional analysis of 261 patients. J. Surg. Oncol..

[16] Hull M.A., Molina G., Niemierko A. (2017). Improved local control with an aggressive strategy of preoperative (with or without intraoperative) radiation therapy combined with radical surgical resection for retroperitoneal sarcoma. J. Surg. Oncol..

[17] Messiou C., Moskovic E., Vanel D. (2017 Jul). Primary retroperitoneal soft tissue sarcoma: imaging appearances, pitfalls and diagnostic algorithm. Eur. J. Surg. Oncol..

